# Performance of a Limiting-Antigen Avidity Enzyme Immunoassay for Cross-Sectional Estimation of HIV Incidence in the United States

**DOI:** 10.1371/journal.pone.0082772

**Published:** 2013-12-27

**Authors:** Jacob Konikoff, Ron Brookmeyer, Andrew F. Longosz, Matthew M. Cousins, Connie Celum, Susan P. Buchbinder, George R. Seage, Gregory D. Kirk, Richard D. Moore, Shruti H. Mehta, Joseph B. Margolick, Joelle Brown, Kenneth H. Mayer, Beryl A. Koblin, Jessica E. Justman, Sally L. Hodder, Thomas C. Quinn, Susan H. Eshleman, Oliver Laeyendecker

**Affiliations:** 1 Department of Biostatistics, School of Public Health, University of California Los Angeles, Los Angeles, California, United States of America; 2 National Institute of Allergy and Infectious Diseases, National Institutes of Health, Bethesda, Maryland, United States of America; 3 Department of Pathology, Johns Hopkins University School of Medicine, Baltimore, Maryland, United States of America; 4 Departments. of Global Health and Medicine, University of Washington, Seattle, Washington, United States of America; 5 Bridge HIV, San Francisco Department of Health, San Francisco, California, United States of America; 6 Departments of Epidemiology and Medicine, UCSF, San Francisco, California, United States of America; 7 Department of Epidemiology, Harvard School of Public Health, Boston, Massachusetts, United States of America; 8 Department of Epidemiology, Johns Hopkins Bloomberg School of Public Health, Baltimore, Maryland, United States of America; 9 Department of Medicine, Johns Hopkins University School of Medicine, Baltimore, Maryland, United States of America; 10 Department of Molecular Microbiology and Immunology, Johns Hopkins Bloomberg School of Public Health, Baltimore, Maryland, United States of America; 11 Department of Epidemiology, School of Public Health, University of California Los Angeles, Los Angeles, California, United States of America; 12 Department of Global Health and Population Harvard School of Public Health, Boston, Massachusetts, United States of America; 13 New York Blood Center, New York, New York, United States of America; 14 Department of Epidemiology, Columbia University Mailman School of Public Health, New York, United States of America; 15 Department of Medicine, New Jersey Medical School, Newark, New Jersey, United States of America; 16 National Institute of Allergy and Infectious Diseases, National Institutes of Health, Bethesda, Maryland, United States of America; University of Athens Medical School, Greece

## Abstract

**Background:**

A limiting antigen avidity enzyme immunoassay (HIV-1 LAg-Avidity assay) was recently developed for cross-sectional HIV incidence estimation. We evaluated the performance of the LAg-Avidity assay alone and in multi-assay algorithms (MAAs) that included other biomarkers.

**Methods and Findings:**

Performance of testing algorithms was evaluated using 2,282 samples from individuals in the United States collected 1 month to >8 years after HIV seroconversion. The capacity of selected testing algorithms to accurately estimate incidence was evaluated in three longitudinal cohorts. When used in a single-assay format, the LAg-Avidity assay classified some individuals infected >5 years as assay positive and failed to provide reliable incidence estimates in cohorts that included individuals with long-term infections. We evaluated >500,000 testing algorithms, that included the LAg-Avidity assay alone and MAAs with other biomarkers (BED capture immunoassay [BED-CEIA], BioRad-Avidity assay, HIV viral load, CD4 cell count), varying the assays and assay cutoffs. We identified an optimized 2-assay MAA that included the LAg-Avidity and BioRad-Avidity assays, and an optimized 4-assay MAA that included those assays, as well as HIV viral load and CD4 cell count. The two optimized MAAs classified all 845 samples from individuals infected >5 years as MAA negative and estimated incidence within a year of sample collection. These two MAAs produced incidence estimates that were consistent with those from longitudinal follow-up of cohorts. A comparison of the laboratory assay costs of the MAAs was also performed, and we found that the costs associated with the optimal two assay MAA were substantially less than with the four assay MAA.

**Conclusions:**

The LAg-Avidity assay did not perform well in a single-assay format, regardless of the assay cutoff. MAAs that include the LAg-Avidity and BioRad-Avidity assays, with or without viral load and CD4 cell count, provide accurate incidence estimates.

## Introduction

HIV incidence measures the rate of new HIV infections in a population [1]. Accurate incidence estimates are needed to identify populations at increased risk of HIV acquisition, monitor the HIV/AIDS epidemic, and evaluate interventions for HIV prevention [2], [3]. Cohort studies that identify HIV seroconverters are expensive and time-consuming, and may not provide reliable incidence estimates because of selection bias, changes in behavior associated with study participation, and loss to follow-up [4]. Unfortunately, serologic assays developed for cross-sectional incidence estimation often overestimate HIV incidence because some long-term infections are classified as assay positive (incident) [5]–[9]. Use of multi-assay algorithms (MAAs) to estimate incidence in cross-sectional surveys is a promising alternative approach for estimating HIV incidence [10], [11]. We recently demonstrated that accurate incidence estimates can be obtained using MAAs that include the BED capture immunoassay (BED-CEIA) [12], the BioRad-Avidity assay [13], and viral load, with or without a second non-serologic biomarker (either CD4 cell count or HIV diversity) [14]–[16].

The United States (US) Centers for Disease Control recently developed a limiting antigen avidity enzyme immunoassay (LAg-Avidity assay) for cross-sectional HIV incidence estimation [17], [18]. This assay measures the avidity of antibody binding to low concentrations of a multi-subtype peptide derived from an immunodominant region of gp41. This assay is commercially available and has been promoted for HIV incidence estimation [19], [20]. However, data from other research groups supporting use of this assay are limited [21], and a recent editorial called for a thorough independent evaluation of the LAg-Avidity assay [22]. The kit manufacturer (SEDIA Biosciences, Portland, OR) recommends excluding individuals with advanced HIV disease (e.g., CD4 cell count <200 cells/mm^3^), elite controllers, and individuals on antiretroviral therapy from incidence surveys [23]. However, no guidance is provided in the package insert on how to combine data from the LAg-Avidity assay and other assays, or what cutoffs should be used for other assays (e.g., HIV viral load). The manufacturer recently recommended increasing the LAg-Avidity assay cutoff from 1.0 to 1.5 normalized optical density units (OD-n) and reducing the mean window period used for incidence estimation from 141 to 130 days [24]; the mean window period is the average time that an individual appears recently infected using an assay or MAA.

In this report, we evaluated the performance of the LAg-Avidity assay alone and in MAAs that included other serologic and non-serologic biomarkers. We evaluated the probability that samples were classified as assay positive or MAA positive over time (using tabulated classification frequencies and fitted smoothed spline curves). We also compared the mean window periods and shadows of the different testing approaches, as well as the relative sample sizes needed for incidence surveys, and relative costs. Finally, we evaluated the ability of the different testing approaches to produce incidence estimates in three cohorts with low, medium, and high incidence that were consistent with those obtained by direct longitudinal follow-up.

## Methods

### Ethics Statement

Written informed consent was obtained from study participants and all studies were reviewed and approved by relevant institutional review boards. Only stored samples from individuals who consented to have their samples could be used for future research were used in this investigation. No new samples were obtained specifically for this study. The study for cross sectional incidence testing on stored study samples was approved by the institutional review board of the Johns Hopkins University. The research was conducted according to the principles expressed in the Declaration of Helsinki.

### Samples used for analysis

The performance of the LAg-Avidity assay and MAAs that include the LAg-Avidity assay was evaluated using 1,782 plasma and serum samples from three cohort studies in the US (duration of infection from 1 month to >8 years): the Multicenter AIDS Cohort Study [25] (MACS, men who have sex with men [MSM], 564 samples, 3 to >8 years after seroconversion), the AIDS Linked to the IntraVenous Experience cohort [26] (ALIVE, persons who inject drugs, 410 samples, 2 to 6 years after seroconversion), and the HIV Network for Prevention Trials 001/001.1 vaccine preparedness cohort [27] (HIVNET 001, men and women with different risk factors for HIV infection, 808 samples, 1 month to 4 years after seroconversion). Further assessments were performed using 500 additional samples from the Johns Hopkins Hospital Clinical Cohort [28] (JHHCC, varied risk factors, approximately one half are persons who inject drugs, all >8 years after seroconversion). Detailed descriptions of these sample sets are provided in previous publications [14], [29]. Two of the 1,782 samples did not have data from the LAg-Avidity assay; those samples were not included in the initial search for optimal testing algorithms (see below), but were included in some of the subsequent analyses. Cross-sectional incidence estimates were generated using additional samples obtained from the HIVNET 001 study (see above) [27], and two other cohort studies from the US: the HIV Prevention Trials Network (HPTN) 061 study (a study of black MSM in the US) [30] and the HPTN 064 study (a study of women with increased HIV acquisition risk from high poverty and HIV prevalence areas in the US) [31].

### Laboratory methods

The LAg-Avidity assay (HIV-1 LAg-Avidity EIA, SEDIA Biosciences Corporation, Portland, OR) was performed according to the manufacturer's instructions. The results are reported as OD-n. Samples with an initial result <2.0 OD-n were retested in triplicate for confirmation and the median confirmation value was used for analysis. Most of the data for the BED-CEIA, the BioRad-Avidity assay, and HIV viral load were generated in previous studies. The BED-CEIA (Calypte Biomedical Corporation, Lake Oswego, OR) was performed according to the manufacturer's instructions [12]. The BioRad-Avidity assay is based on the Genetic Systems 1/2+O ELISA [13] (Bio-Rad Laboratories, Redmond, WA), using diethyl amine as the chaotropic agent with the following modification: the diethyl amine was diluted in water and the initial incubation time was decreased to 30 minutes. CD4 cell count data were obtained in the cohort studies.

### Statistical Methods

Samples analyzed using the LAg-Avidity assay alone were classified as assay positive (below the assay cutoff) or assay negative (above the assay cutoff); similarly, samples analyzed using MAAs were classified as MAA positive (meeting the criteria of all component assays) or MAA negative (failing to meet the criteria of one or more of the component assays). Samples classified as assay positive or MAA positive are counted as incident infections in incidence estimates.

In the first phase of the analysis, we calculated the number of samples classified as assay positive or MAA positive by duration of infection (years after HIV seroconversion). For individuals who had acute or early HIV infection at the time of sample collection (Fiebig stage I or II [32]), seroconversion was assumed to have occurred within 28 days after sample collection. For other individuals, seroconversion was assumed to have occurred between the last negative and first positive HIV tests. For these individuals the median number of days between last negative and first positive HIV tests was 184 days (interquartile range: 168–201 days). We first assessed performance of: (1) the LAg-Avidity assay alone (assay cutoffs: 0.5, 1.0, 1.5, or 3.0 OD-n), (2) a 3-assay MAA that included the LAg-Avidity assay (assay cutoff: 1.5 OD-n) with viral load (assay cutoff: 1,000 copies/mL) and CD4 cell count (assay cutoff: 200 cells/mm^3^), and (3) 2-assay MAAs that included the LAg-Avidity assay (assay cutoffs of 1.0 or 1.5 OD-n) and viral load (assay cutoff: 1,000 copies/mL) without CD4 cell count. For these analyses, duration of infection was classified into time intervals by midpoint imputation.

The proportion of samples that were classified as assay positive or MAA positive was also assessed as a function of duration of infection by fitting logistic regression models with cubic splines [14]. Seroconversion dates were sampled from uniform distributions over the potential seroconversion intervals defined above. The mean window period (i.e., mean duration a person is assay or MAA positive) and the shadow (a measure of how far back in time incidence is being estimated [32], [33]) were estimated by using multiple imputation and averaging the results from the fitted curves [14], [33], [34]. The mean window period and shadow were not calculated for an assay or MAA if the probability of a positive result did not converge to zero (<0.001) by 8 years after seroconversion. To account for multiple samples from the same individual, confidence intervals (CIs) were calculated using bootstrapping, blocking on individuals (so that all samples from the same individual were included in each bootstrapped sample). The bootstrap was stratified by cohort study [14], [33], [34].

The second phase of the analysis further assessed the performance of the LAg-Avidity assay alone and MAAs that included other serologic and non-serologic assays to determine optimal algorithms based on criteria described below. Over 30,000 testing algorithms were considered by analyzing all possible combinations of the following cutoffs for the component assays: LAg-Avidity assay: 22 cutoffs ranging from 0.5–3.9 OD-n; BioRad-Avidity assay: 12 cutoffs ranging from 30–100% (avidity index); viral load: 10 cutoffs ranging from 400–10,000 copies/mL; CD4 cell count: 12 cutoffs ranging from 50–1,000 cells/mm^3^. The goal of this analysis was to evaluate different cutoffs for the LAg-Avidity assay alone, and to identify MAAs with optimal assay combinations and assay cutoffs [14]. Testing algorithms were evaluated further if they met the following requirements: (1) the estimated probability of being classified as assay or MAA positive 8 years after seroconversion was <0.001 (based on analysis of samples from the MACS, ALIVE, and HIVNET 001 cohorts), (2) all 500 samples from the JHHCC (all infected >8 years) were classified as assay or MAA negative, (3) the shadow was <250 days and the upper 95% confidence limit of the shadow was <1 year. For each class of testing algorithms that met these requirements, we identified and deemed optimal the assay or MAA that had the largest mean window period, as this minimizes the variability in incidence estimates [33]. We then expanded the search to >500,000 algorithms that included all combinations of the assays and cutoffs listed above, along with the BED-CEIA (using 16 cutoffs for the BED-CEIA ranging from 0.4 to 1.8 OD-n).

In the third phase of the analysis, we compared the number of samples needed in cross-sectional surveys to obtain the same precision in incidence estimates using two optimized MAAs (relative sample size, calculated as the ratio of the mean window periods). We also compared the relative cost of the two MAAs, optimizing the order of the component assays. For this analysis, the relative costs of the LAg-Avidity assay, BioRad-Avidity assay, CD4 cell count, and viral load were considered to be r, r, 2.5r, and 5r, respectively, where r is the unit cost of the LAg-Avidity assay. For the cost analysis, CD4 cell count was listed first when used in a MAA, since CD4 cell count must be performed at the time of sample collection. In addition, costs were adjusted by multiplying the relative costs by the relative sample sizes that are needed to account for differences in mean window periods. These analyses considered only the cost of sample testing.

In the last phase of the analysis, we compared cross-sectional incidence estimates obtained using the LAg-Avidity assay and MAAs to longitudinal incidence estimates previously reported for three cohort studies from the US with varying HIV incidence. Cross-sectional incidence estimates were calculated for: (1) the LAg-Avidity assay alone (using assay cutoffs of 1.0 or 1.5 OD-n), (2) the LAg-Avidity assay with viral load and CD4 cell count (using assay cutoffs of 1.5 OD-n, 1,000 copies/mL, and 200 cells/mm^3^, respectively), and (3) two optimized MAAs. These analyses were performed for the following three cohorts: HIVNET 001 (see above), HPTN 061 (a cohort of black MSM) [30], and HPTN 064 (a cohort of women in high poverty areas at increase risk of HIV acquisition) [31]. For these analyses, cross-sectional incidence estimates were calculated as: [(number of samples classified as assay or MAA positive)×(100)]/[(number of HIV-negative samples)×(mean window period in years)]. The CIs calculated for these estimates accounted for uncertainty in the mean window period [35].

### Human subjects

The cohort studies described in this report were conducted according to the ethical standards set forth by the institutional review boards of the participating institutions and the Helsinki Declaration of the World Medical Association; participants provided written informed consent. The work described in this report involved analysis of stored samples and data from those studies. No participants were recruited or followed in the course of this work. This research was approved by the Institutional Review Board of the Johns Hopkins University.

## Results

### Performance of the LAg-Avidity assay used in a single-assay format

We first calculated the proportion of samples that were classified as assay positive by duration of infection using the Lag-Avidity assay alone, using four different assay cutoffs (Table 1). Using the cutoff of 1.0 OD-n that was originally recommended for this assay [23], 32% of samples from individuals infected <6 months were classified as assay positive, while 3% of samples from individuals infected >5 years were classified as assay positive. Raising the assay cutoff to 1.5 OD-n (the cutoff currently recommended by the manufacturer [24]) increased the percentage of samples from individuals infected <6 months that were classified as assay positive (from 32% to 43%), but also increased the percentage of samples from individuals infected >5 years who were classified as assay positive (from 3% to 5%). When the assay cutoff was increased to 3.0 OD-n, a greater proportion of samples from individuals infected <6 months were classified as assay positive (74%), but a much larger proportion of samples from individuals infected >5 years were classified as assay positive (15%). When the assay cutoff was lowered to 0.5 OD-n, the percentage of individuals infected >5 years classified as assay positive dropped to 2%; however, at this stringent cutoff, only 13% of samples from individuals infected <6 months were classified as MAA positive.

**Table 1:** Number of samples classified as assay positive using the LAg-Avidity assay alone.

Duration of infection		Lag-Avidity assay cutoff			
(years)	N	<0.5	<1.0	<1.5	<3.0
0.0 to <0.5	142	18 (13%)	46 (32%)	61 (43%)	105 (74%)
0.5 to <1.0	167	8 (5%)	17 (10%)	36 (22%)	75 (45%)
1.0 to <2.0	262	20 (8%)	25 (10%)	35 (13%)	90 (34%)
2.0 to <3.0	301	21 (7%)	28 (9%)	34 (11%)	69 (23%)
3.0 to <4.0	440	10 (2%)	17 (4%)	23 (5%)	64 (15%)
4.0 to <5.0	125	1 (1%)	5 (4%)	11 (9%)	15 (12%)
≥5.0	343	7 (2%)	10 (3%)	18 (5%)	51 (15%)

Samples from the MACS, ALIVE, and HIVNET 001 cohorts (N = 1,780) were tested using the LAg-Avidity assay (LAg). Four assay cutoffs were evaluated: 0.5, 1.0, 1.5, and 3.0 optical density units (OD-n); samples were classified as assay positive if they were below the assay cutoff. The number and percentage of samples that were assay positive are presented separately for individuals with different durations of HIV infection (see Methods). N indicates the number of samples in each group.

**Figure 1:**
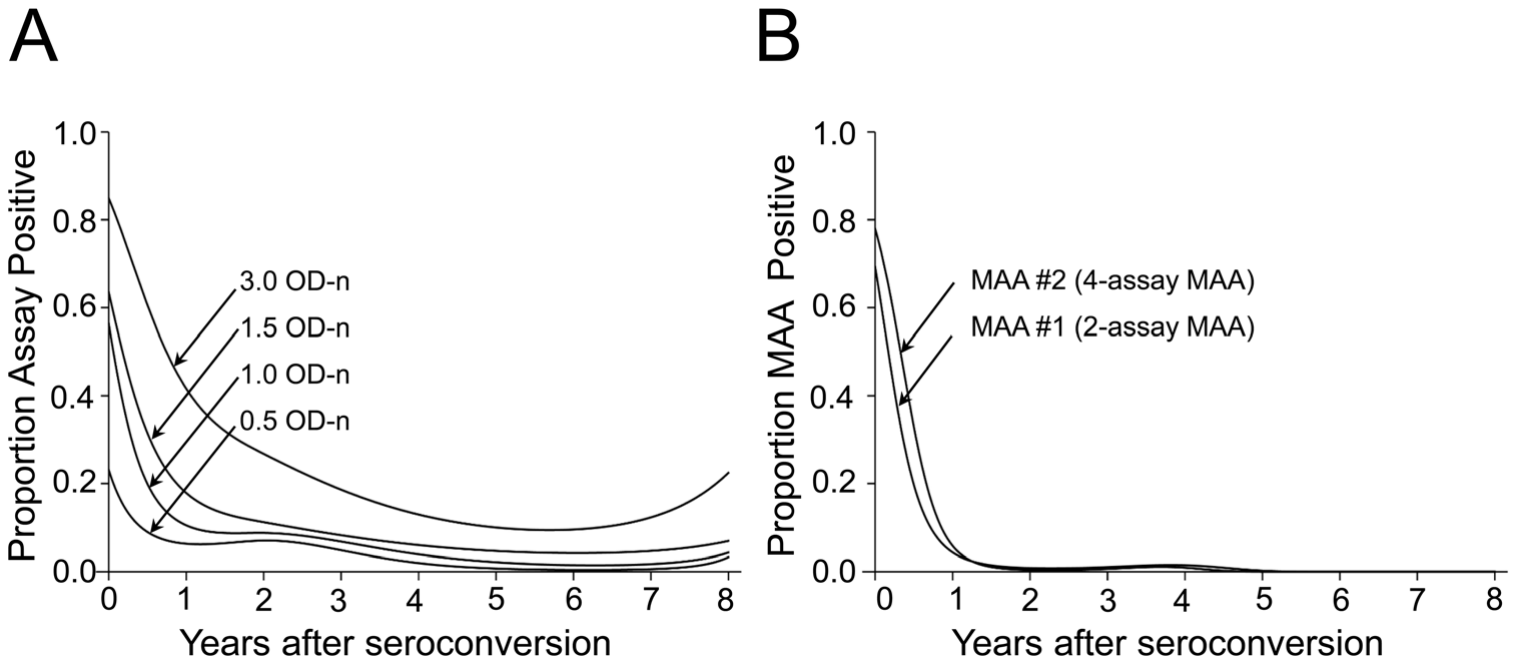
Proportion of samples classified as assay positive using the LAg-Avidity assay alone or with HIV viral load, as a function of the duration of HIV infection. Probability curves were generated by analyzing samples from three cohort studies (see Methods). (A) Probability curves generated using the LAg-Avidity assay with four different assay cutoffs (0.5, 1.0, 1.5, and 3.0 normalized optical density units [OD-n]). Samples were classified as assay positive if the LAg-Avidity assay result was below the assay cutoff. (B) Probability curves generated using the MAAs shown in Figure 2. Samples were classified as MAA positive if results from each of the component assays met the requirements of the MAA.

The proportion of individuals classified as assay positive using the LAg-Avidity assay alone was also analyzed by fitting probability models with cubic splines (Figure 1A). These probability models did not approach 0% assay positive by 8 years using any of the four cutoffs; this indicates that the assay continued to classify some individuals as assay positive 8 years after seroconversion. We did not calculate mean window periods or shadows for the LAg-Avidity assay using any of these assay cutoffs, because of the persistent classification of samples from individuals with long-term HIV infection as assay positive.

### Performance of the LAg-Avidity assay in MAAs that included HIV viral load, with or without CD4 cell count

The package insert included with the LAg-Avidity assay recommends excluding individuals with advanced HIV disease (CD4 cell count <200 cells/mm^3^), elite controllers, and individuals on antiretroviral therapy. To address this, we evaluated the performance of the LAg-Avidity assay in a 3-assay MAA that included viral load and CD4 cell count (Table 2). In contrast to the LAg-Avidity assay alone, this MAA did not classify any samples from individuals infected >5 years as MAA positive and had a shadow <1 year (158 days). Our analysis indicated that this MAA had a mean window period of only 85 days, which is lower than the mean window period of 130 days (95% CI: 118–142) provided in the current package insert [24].

**Table 2:** Performance characteristics of MAAs that include the LAg-Avidity assay and HIV viral load, with and without CD4 cell count.

Duration of infection (years)	N	LAg <1.0 VL >1,000	LAg <1.5 VL >1,000	CD4 >200 LAg <1.5 VL >1,000
0.0 to <0.5	142	32 (23%)	46 (32%)	45 (32%)
0.5 to <1.0	167	4 (2%)	13 (8%)	13 (8%)
1.0 to <2.0	262	3 (1%)	3 (1%)	0 (0%)
2.0 to <3.0	301	4 (1%)	4 (1%)	2 (1%)
3.0 to <4.0	440	2 (<1%)	4 (1%)	1 (<1%)
4.0 to <5.0	125	3 (2%)	5 (4%)	1 (1%)
≥5.0	343	1 (<1%)	4 (1%)	0 (0%)
Mean Window Period		80 days	134 days	85 days
Shadow		446 days	690 days	158 days

Samples from the MACS, ALIVE, and HIVNET 001 cohorts (N = 1,780) were tested using MAAs that included the LAg-Avidity assay and HIV viral load, with and without CD4 cell count. The cutoffs used for the LAg-Avidity assay (1.0 or 1.5 normalized optical density units [OD-n]) and the cutoff used for CD4 cell count (200 cells/mm^3^) are recommended by the assay manufacturer. The cutoff used for HIV viral load (VL, 1,000 copies/mL) was previously suggested for use with the Lag-Avidity assay along with self-report of antiretroviral treatment [35]. Samples were classified as MAA positive if they met the criteria of each component assay. In the table, CD4 cell count testing is listed first in the MAA, since that testing must be performed at the time of sample collection. The number and percentage of samples that were MAA positive are presented separately for individuals with different durations of HIV infection (see Methods). N indicates the number of samples in each group. The mean window period and shadow for each MAA are shown.

For comparison, we evaluated the performance of the LAg-Avidity assay in 2-assay MAAs that included viral load without CD4 cell count (Table 2). For this analysis, we evaluated two MAAs that included the LAg-Avidity assay (using an assay cutoff of 1.0 or 1.5 OD-n) with viral load (assay cutoff: of 1,000 copies/mL). A viral load cutoff of 1,000 copies/mL was used previously by the developers of the LAg-Avidity assay to identify individuals with viral suppression [36]. These 2-assay MAAs classified 2–4% of the samples from individuals infected for 4–5 years and some individuals infected >5 years as MAA positive. In contrast to the LAg-Avidity assay alone (Figure 1), these two MAAs did converge to 0% MAA positive by 8 years (data not shown). These MAAs had mean window period of 80 and 134 (using LAg-avidity assay cutoffs of 1.0 and 1.5, respectively). However, the shadows for these MAAs were >1 year (446 and 690 days respectively), indicating that these MAAs were estimating incidence over more than a year prior to sample collection. Therefore, these MAAs were not evaluated further.

### Comparison of the performance of optimized MAAs

**Figure 2:**
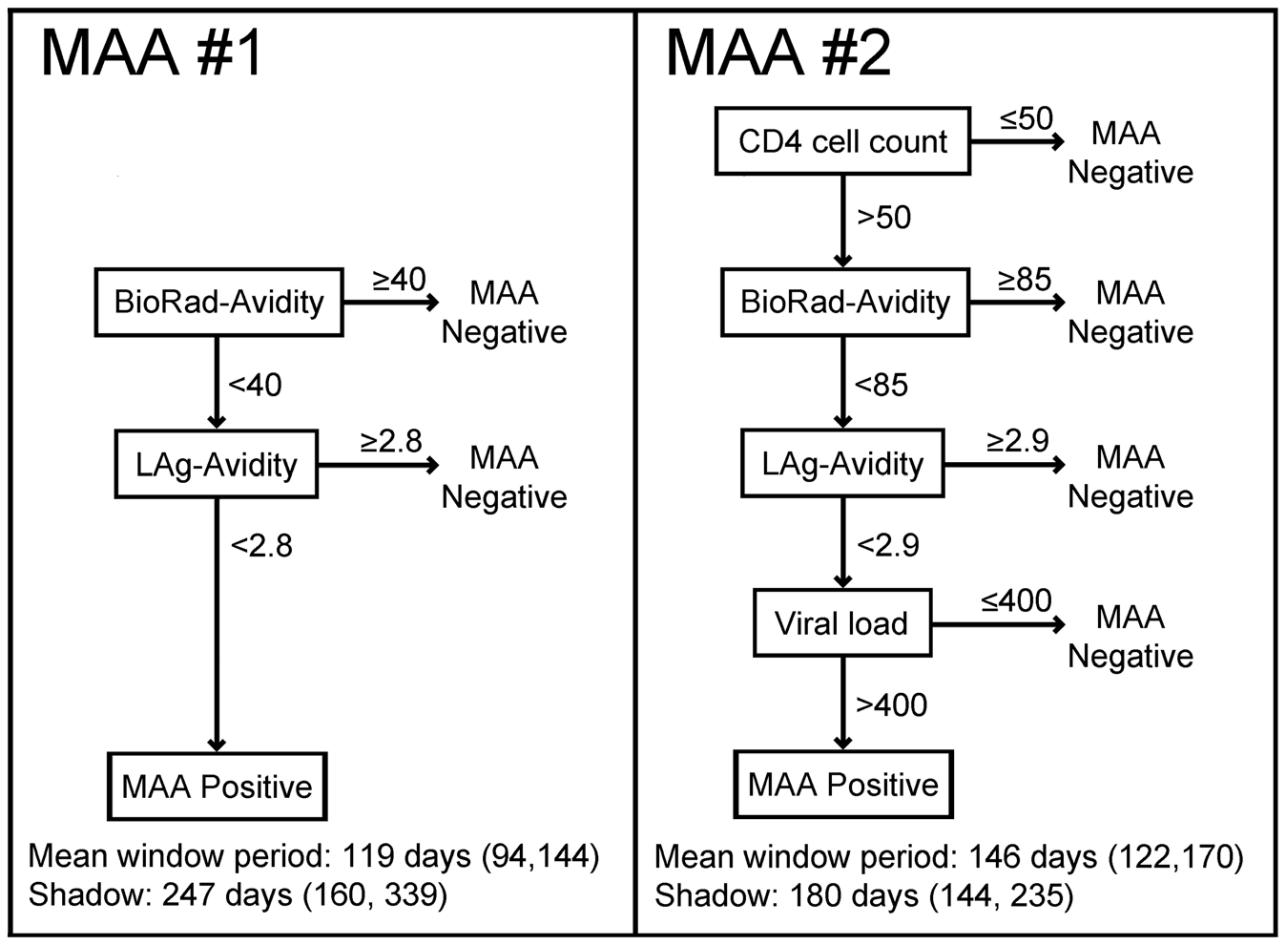
Optimized multi-assay algorithms (MAAs). The figure shows two optimized MAAs that include the LAg-Avidity assay. The assays, assay cutoffs, window periods, and shadows are shown for each MAA. The 95% confidence intervals for the window periods and shadows are shown in parentheses. Abbreviations: LAg-Avidity: limiting antigen (LAg) avidity assay; BioRad-Avidity: BioRad-Avidity assay; OD-n: normalized optical density. The following units are used for the component assays: LAg-Avidity assay: OD-n; BioRad-Avidity assay: percentage (avidity index); CD4 cell count: cells/mm^3^; viral load: copies/mL.

The next step in the analysis was to compare the performance of >30,000 testing algorithms that included the LAg-Avidity assay alone or in combination with up to three other assays, using a range of cutoffs for each assay (see Methods). For each class of testing algorithms that met pre-set requirements (see Methods), we identified the MAA that had the largest mean window period (referred to as optimized MAAs). None of the algorithms using the LAg-Avidity assay alone or the LAg-Avidity assay with viral load met pre-set performance requirements (see Methods). The optimized 2-assay MAA included the LAg-Avidity and BioRad-Avidity assays (MAA #1, Figure 2); this MAA had a mean window period of 119 days. The optimized 4-assay MAA included the LAg-Avidity assay, the BioRad-Avidity assay, CD4 cell count, and viral load (MAA #2, Figure 2); this MAA had a mean window period of 146 days and was the top-performing MAA among >30,000 algorithms evaluated. Interestingly, our search did not identify a 3-assay MAA that was superior to either the optimized 2-assay MAA or the optimized 4-assay MAA (considering mean window period and relative cost).

Because the target antigens used in the LAg-Avidity assay and BED-CEIA are similar [12], [17], we did not include the BED-CEIA in our initial analyses. When we expanded the search to include the BED-CEIA, the best MAA identified (from among >500,000 MAAs evaluated) included the BED-CEIA (assay cutoff 1.6 OD-n), the BioRad-Avidity assay (assay cutoff 85%), CD4 cell count (assay cutoff: 200 cells/mm^3^), and viral load (assay cutoff: 400 copies/mL); this MAA is identical to MAA #2 (Figure 2), except that the LAg-Avidity assay is replaced with the BED-CEIA. Because the 4-assay MAA that includes the BED-CEIA is identical to the optimized 4-assay MAA described in a previous report [14], we did not perform any additional analyses with that MAA.

Performance characteristics of the optimized 2-assay and 4-assay MAAs (MAA #1 and MAA #2, Figure 2) are shown in Table 3. Probability curves for these MAAs are shown in Figure 1B. The proportion of samples identified as MAA positive converged to zero for both MAAs, and none of the 845 samples from individuals infected >5 years was classified as MAA positive (Table 3). Because the 2-assay MAA has a shorter mean window period than the 4-assay MAA (119 days vs. 146 days), larger cross-sectional surveys would be required to obtain incidence estimates with the same precision (relative sample size: 123% compared to MAA #2).

**Table 3:** Performance characteristics of optimized MAAs that include the LAg-Avidity assay.

		MAA #1	MAA #2
		–	CD4 >50
Duration of infection		BioRad <40	BioRad <85
(years)	N	LAg <2.8	LAg <2.9
		–	VL >400
0.0 to <0.5	142	57 (40%)	76 (53%)
0.5 to <1.0	167	8 (5%)	24 (14%)
1.0 to <2.0	262	6 (2%)	4 (2%)
2.0 to <3.0	301	4 (1%)	2 (1%)
3.0 to <4.0	440	4 (1%)	3 (1%)
4.0 to <5.0	125	2 (2%)	1 (1%)
5.0 to <8.0	333	0 (0%)	0 (0%)
≥8.0	512	0 (0%)	0 (0%)
Mean Window Period		119 (94, 144)	146 (122, 170)
Shadow		247 (160, 339)	180 (144, 235)
Relative sample size		123%	100%
Relative cost		25%	100%
Adjusted relative cost		31%	100%

Samples from the MACS, ALIVE, and HIVNET 001 cohorts (N = 1,782) and samples from the JHHCC cohort (N = 500) were tested using MAAs that included the LAg-Avidity assay (MAA #1 and MAA #2, Figure 2). The following units were used for the component assays: LAg-Avidity assay, normalized optical density units (OD-n); BioRad-Avidity assay, percentage (avidity index); viral load, copies/mL; CD4 cell count, cells/mm^3^. Samples were classified as MAA positive if they met the criteria for all of the assays in the MAA (Figure 2). The number and percentage of samples that were MAA positive are presented separately for individuals with different durations of HIV infection (see Methods). N indicates the number of samples in each group. The following additional performance measures are shown: mean window period, shadow, relative sample size needed for cross-sectional surveys (with MAA #2 as a reference), relative cost, and relative cost adjusted for the mean window period (with MAA #2 as a reference) (see Methods). The cost analyses assumed that assays would be performed in the order shown.

### Relative cost of optimized MAAs

We compared the relative cost of sample analysis using the optimized 2-assay and 4-assay MAAs (Table 3, see Methods). Relative cost was also adjusted for the mean window period of each MAA, to account for differences in the sample sizes that would be needed to obtain incidence estimates with similar precision. This analysis assumed that testing would be performed using the assays in the order shown in Figure 2. The cost of the 2-assay MAA was less than a third of the cost of the 4-assay MAA, even after adjusting for the shorter mean window period.

Of note, one of the MAAs evaluated in the initial search of over >30,000 algorithms was the 3-assay MAA described above that included the LAg-Avidity assay (cutoff <1.5 OD-n), viral load (cutoff >1,000 copies/mL) and CD4 cell count (cutoff >200 cells/mm^3^, Table 2) [24]. The implication of the lower mean window period (85 vs. 130 days) is that an incidence survey would have to be 72% larger using this MAA than one using the optimized 4-assay MAA (mean window period 146 days), and 40% larger than one using the optimized 2-assay MAA (mean window period 119 days). Furthermore, the adjusted relative cost of this 3-assay MAA (using a window period of 85 days) was greater than the costs of both the optimized 2 and 4-assay MAAs, which makes it undesirable. Even if the manufacturer's recommended mean window period of 130 days were used to adjust the relative cost of the 3-assay MAA, it would still be more costly than both of the optimized MAAs (adjusted relative cost: 342% compared to the optimized 2-assay MAA, 106% compared to the optimized 4-assay MAA).

### Comparison of incidence estimates obtained using optimized MAAs to those obtained from cohort follow-up

As a final step in our analysis, we used the LAg-Avidity assay alone and selected MAAs to generate cross-sectional incidence estimates for three cohorts: HPTN 064, HIVNET 001, and HPTN 061 (see Methods, Table 4). These incidence estimates were compared to longitudinal incidence based on cohort follow-up [30], [31], [37]. When the LAg-Avidity assay was used alone with a cutoff of 1.0 or 1.5 OD-n (cutoffs recommended by the manufacturer), the 95% CIs of the incidence estimates did not cover the longitudinal incidence point estimates for one of the three cohorts (HPTN 061). In contrast, the 95% CIs for all three of the MAAs evaluated covered the longitudinal incidence point estimates for all three cohorts. In addition, the incidence estimates obtained using the MAAs were much closer to the longitudinal estimates than the incidence estimates obtained using the LAg-Avidity assay alone for the two cohorts that included individuals with long-term infection (i.e., individuals who were HIV infected at enrollment, HPTN 064 and HPTN 061, % difference, Table 4). The optimized 4-assay MAA provided incidence estimates that were closest to the longitudinal incidence estimates for all three cohorts (cross-sectional vs. longitudinal estimates: HPTN 064: 0.26% vs. 0.24%; HIVNET 001: 1.09% vs. 1.04%; HPTN 061: 3.44% vs. 3.02%).

**Table 4:** HIV incidence estimates for three clinical cohorts in the United States.

Method used to estimate incidence	Window period		HPTN 064^a^	HIVNET 001	HPTN 061^b^
Longitudinal	–	Follow-up period (months)	0–12	12–18	0–12
		# seroconverters	4	24	28
		Person-years follow-up	1,639	2,304	926
		Observed incidence	0.24% (0.07, 0.62)	1.04% (0.70, 1.55)	3.02% (2.01, 4.37)
Cross-sectional	–	Visits analyzed (months)	6–12	18	12
		# HIV positive^c^	33	90	246
		# HIV negative	1,947	4,175	872
		Study visit (months)	(12)	(18)	(12)
		# tested	33	79	246
LAg-Avidity <1.0	141 days^d^	# assay positive	4	12	20
		Incidence estimate	0.53% (0.07, 1.39)	0.85% (0.43, 1.52)	5.94% (3.56, 9.45)
		% difference	121%	−18%	97%
LAg-Avidity <1.5	130 days^d^	# assay positive	4	15	29
		Incidence estimate	0.58% (0.16, 1.49)	1.15% (0.64, 1.92)	9.34% (6.20, 13.59)
		% difference	142%	11%	209%
LAg-Avidity <1.5 +	130 days^d^	# MAA positive	2	8	8
viral load >1,000 +		Incidence estimate	0.29% (0.03, 1.05)	0.61% (0.26, 1.22)	2.58% (1.11, 5.12)
CD4 >200		% difference	21%	−41%	−15%
MAA #1	119 days^e^	# MAA positive	2	11	13
		Incidence estimate	0.32% (0.04, 1.17)	0.92% (0.45, 1.73)	4.57% (2.37, 8.24)
		% difference	33%	−12%	51%
MAA #2	146 days^e^	# MAA positive	2	16	12
		Incidence estimate	0.26% (0.03, 0.95)	1.09% (0.60, 1.84)	3.44% (1.75, 6.20)
		% difference	8%	5%	14%

The table shows cross-sectional and longitudinal incidence estimates for three clinical cohorts (see Methods); 95% confidence intervals are shown in parentheses. Longitudinal incidence estimates were calculated as the number of seroconversion events divided by the number of person-years of follow-up; these estimates were reported previously [26], [29]–[30]. The % difference is defined as: 100× (the absolute value of the difference between the longitudinal incidence estimate and the cross-sectional incidence estimate) divided by the longitudinal incidence estimate. Cross-sectional incidence estimates were obtained using five testing approaches (the original LAg-Avidity protocol, the revised LA-Avidity protocol, the 3-assay MAA in Table 2, and MAA #1 and MAA #2, described in Figure 2). The following units were used for the component assays: LAg-Avidity assay: normalized optical density units (OD-n); BioRad-Avidity assay: percentage (avidity index); viral load: copies/mL; CD4 cell count: cells/mm^3^.

^a^ In HPTN 061, some study participants who contributed to the longitudinal incidence estimate did not complete the 12-month study visit or did not have a sample stored at that visit and were not included in the cross-sectional incidence estimates.

^b^ In HPTN 064, the primary study outcome was overall HIV incidence, measured as a composite incidence estimate that took into account cross-sectional incidence at enrollment (estimated using a MAA), acute infections at enrollment, and observed incidence during longitudinal follow-up (based on HIV seroconversion). The overall HIV incidence in the HPTN 064 study was 0.32% (95% CI: 0.14–0.74%) [30]. In that study, cross-sectional incidence was assessed using a MAA that included the BED-CEIA, the BioRad-Avidity assay, CD4 cell count, and HIV viral load [43].

^c^ For HPTN 064: A total of 38 women were identified with HIV-infection in the HPTN 064 study. The 33 HIV-positive women included in the cross-sectional incidence assessment at 6–12 months included 28 women who were seropositive at study enrollment, one woman who had acute HIV infection at enrollment, and four women who acquired HIV infection during the study. For HIVNET 001: All participants included in the cross-sectional incidence assessment were HIV-uninfected at study enrollment. For HPTN 061: The 246 men included in the cross-sectional incidence assessment at 12 months included 218 men who were seropositive at study enrollment, three men who had acute HIV infection at enrollment, and 25 men who acquired HIV infection during the study.

^d^ Incidence estimates using these testing approaches were calculated using window periods recommended by the manufacturer of the LAg-assay.

^e^ Incidence estimates using the two optimized MAAs (MAA #1 and MAA #2) were calculated using window periods determined in this report (Table 3).

## Discussion

This report includes a comprehensive evaluation of the performance of the LAg-Avidity assay alone and MAAs that include the LAg-Avidity assay. When used in a single-assay format, the LAg-Avidity assay classified a smaller proportion of samples from individuals with long-term infection as assay positive than the BED-CEIA alone (based on comparison to results previously obtained for the BED-CEIA using the same sample sets [14]). However, the LAg-Avidity assay still classified some individuals with long-term infection as assay positive and failed to provide accurate HIV incidence estimates in cohorts that included individuals with long-term infections (HPTN 064 and HPTN 061). Some individuals who were infected >5 years were also classified as MAA positive when the LAg-Avidity assay was used in 2-assay MAAs that also included viral load. Furthermore, MAAs that included only the LAg-Avidity assay and viral load had shadows that were >1 year, indicating that they were estimating incidence more than a year prior to sample collection.

Using an objective, statistical approach to evaluate >30,000 different testing algorithms that included the LAg-Avidity assay, we identified an optimized 2-assay MAA and an optimized 4-assay MAA. Both of these MAAs include the LAg-Avidity assay and the BioRad-Avidity assay; the 4-assay MAA also includes CD4 cell count and viral load. Both of the optimized MAAs classified all 845 samples from individuals infected >5 years as MAA negative. Furthermore, both of the optimized MAAs provided cross-sectional incidence estimates that were similar to those obtained by longitudinal follow-up for three cohorts in the US with varying HIV incidence. The 4-assay MAA was the top-performing MAA among >30,000 MAAs that did not include the BED-CEIA; this MAA provided incidence estimates that were nearly identical to those obtained by cohort follow-up. Of note, the performance of the optimized 4-assay MAA described in this report was very similar to that of an optimized 4-assay MAA which was the best MAA in the expanded search of >500,000 algorithms; that MAA included the BED-CEIA rather than the LAg-Avidity assay (described previously [14], [16]). These two MAAs use the same cutoffs for the BioRad-Avidity assay, CD4 cell count, and viral load.

Acceptable incidence estimates were also obtained using a 3-assay MAA that included the LAg-Avidity assay, CD4 cell count, and viral load. The cutoffs used for the LAg-Avidity assay (1.5 OD-n) and CD4 cell count (200 cells/mm^3^) in this MAA are recommended by the manufacturer [24], the cutoff used for viral load (1,000 copies/mL) was used by the developers of the LAg-Avidity assay in a previous study (in conjunction with self-reported use of antiretroviral therapy (ART) [36]. We did not include self-report of ART in our algorithms; recent reports indicate that self-report of ART use is often unreliable [38]–[40]. This 3-assay MAA did not classify any of samples from individuals infected >5 years as MAA positive and had a shadow <1 year. However, our analysis indicated that this MAA had a mean window period of only 85 days, which is shorter than the mean window period of 130 days (95% CI: 118–142) provided in the current package insert [24] and is shorter than the mean window periods of the optimized 2-assay and 4-assay MAAs (119 and 146 days, respectively). The implication of the lower mean window period is that a cross sectional survey to measure incidence would have to be larger using the 3-assay MAA than one using either the optimized 2-assay or the optimized 4-assay MAA. Furthermore, the adjusted relative cost for sample testing using this 3-assay MAA was greater than the cost for sample testing using the optimized 4-assay MAA, which makes it difficult to justify its use.

We recognize that the confidence intervals for incidence obtained using the MAAs (which incorporate uncertainty in the mean window period) are larger than those obtained by longitudinal follow-up of cohorts. Additional studies that improve the precision of the mean window period would reduce the width of these confidence intervals. The width of the confidence intervals could also be reduced with larger cross-sectional surveys. Furthermore, even with larger cross-sectional surveys, it may be less expensive to estimate incidence using a MAA than a cohort study because no longitudinal follow-up is required.

Our identification of an optimized 2-assay MAA that does not require CD4 cell count or HIV viral load has major implications for global HIV surveillance. The optimized 2-assay MAA identified in this report, which includes the LAg-Avidity and BioRad-Avidity assays without CD4 or viral load, has lower testing costs than the optimized 4-assay MAA, even after accounting for the larger sample sizes that would be needed with the 2-assay MAA because of its shorter mean window period. Note that this cost comparison only included costs for laboratory testing; costs associated with obtaining samples for analysis were not included in this analysis. Because the 2-assay MAA does not require CD4 cell count data, it can be performed entirely using stored plasma or serum samples, which would be an advantage in some studies. We previously described an optimized 3-assay MAA that included the BED-CEIA, the BioRad-Avidity assay, and viral load (without CD4 cell count) [14]; an advantage of the 2-assay MAA described in this report over the 3-assay MAA described in the previous report [14] is that the 2-assay MAA also does not require viral load testing. Furthermore, recent data suggests that both the LAg-Avidity and BioRad-Avidity assays can be performed using dried blood spots [18], [41]. This could significantly reduce the complexity and cost of sample acquisition, shipping and storage in incidence surveys.

The samples used in this report were obtained from diverse cohorts that included men and women of different ethnicities and ages; and individuals infected through heterosexual, homosexual and parenteral routes. All of these samples were from the US where the most prevalent HIV-1 subtype is B. Serologic assays developed for cross-sectional incidence estimation have been shown to perform differently depending on the infecting subtype [7], [42], [43]. Therefore, additional studies are needed to evaluate the performance of the LAg-Avidity assay (alone and in MAAs) in populations where other HIV-1 subtypes are prevalent.
